# Lady Windermere Syndrome: A Rare Differential Diagnosis for Hemoptysis

**DOI:** 10.7759/cureus.59272

**Published:** 2024-04-29

**Authors:** Manojkumar Krishnan, KVC Janaka, Hiruni Fernando, Dimithri Gamaarachchi, Isuru Perera

**Affiliations:** 1 Internal Medicine, Sri Jayewardenepura General Hospital, Colombo, LKA

**Keywords:** nontuberculous mycobacteria (ntm), hemoptysis, bronchiectasis, mycobacterium avium complex (mac), lady windermere syndrome

## Abstract

Mycobacterium avium complex (MAC) is often observed in immunocompromised individuals. However, when pulmonary MAC infection occurs in immunocompetent individuals, particularly elderly females, characteristically involving the middle lobe and lingula lobe of the lung, it is known as Lady Windermere syndrome (LWS). A 64-year-old female patient with no significant comorbidities presented with a history of low-grade intermittent fever and dry cough for one-month duration complicated with hemoptysis for two days. Her initial investigations and imaging were negative, except for the high-resolution CT (HRCT) finding of bronchiectasis involving the middle lobe and lingula lobe suggestive of MAC infection, which was further confirmed by positive sputum culture for MAC. LWS is a condition that is rarely encountered in clinical settings and seldom described in the literature. Especially in resource-limited settings, arriving at a diagnosis is further hindered by the scarce availability of advanced imaging such as HRCT. In clinical settings where pulmonary tuberculosis is endemic, the differentiation of the two conditions is of paramount importance as the treatment regimens for the two conditions are quite different.

## Introduction

Mycobacterium avium complex (MAC) organisms fall under the nontuberculous mycobacteria (NTM) species that are commonly isolated from environmental sources and are not obligate pathogens. Even though it is not as common as tuberculosis, MAC is the most common cause of NTM infection and has a predisposition to cause lung infections predominantly [1].

Lady Windermere syndrome (LWS) was first described in 1992 in a case series of six cases of MAC infection in immunocompetent elderly females who did not have any preceding chronic lung conditions [2]. This finding was noteworthy because MAC infection is usually known to occur in individuals with preexisting lung diseases or in immunocompromised patients [3]. In the same case series, it was also noted that in these individuals, the MAC infection followed a specific lung involvement pattern where there was isolated middle lobe and lingula lobe involvement with a nodular bronchiectatic pattern [2].

Since the publication of this index case report, several other case reports have been published with MAC lung infections that follow a similar lung involvement pattern occurring primarily in immunocompetent elderly females. In the index case report, it was postulated that the specific lung involvement pattern could probably be because of voluntary cough suppression in the described population of patients [2]. Over the years, other possible associations including thoracic skeletal abnormalities such as pectus excavatum and connective tissue diseases have also been noted in patients diagnosed with LWS [4].

## Case presentation

A previously healthy 64-year-old female patient presented to the hospital with mild intermittent fever for one-month duration complicated with hemoptysis over the last two days. It was a low-grade intermittent fever without a specific fever pattern, and she did not complain of night sweats. The fever was associated with a dry cough and intermittent episodes of pleuritic-type chest pain. Over a period of one month, she had only taken treatment twice as an outpatient and had not been investigated before admission.

She developed hemoptysis two days before admission to the hospital. Initially, she coughed up mildly blood-stained sputum but later on, the hemoptysis worsened where she coughed up about 10 mL of blood on three occasions, prompting her to get admitted to the hospital.

She was a nonsmoker and did not have any significant past medical history nor any history of recurrent lung infections. She had undergone laparoscopic cholecystectomy previously, but apart from that, her past surgical history was unremarkable.

On examination, she was thinly built with a weight of 45 kg and a BMI of 18.1 kg/m^2^. She was afebrile and not pale. Her blood pressure was 140/96, her pulse rate was 80 beats per minute (bpm), and there were no audible murmurs. She had vesicular breathing with a respiratory rate of 17, and her lung examination revealed only occasional wheezing and crepitations scattered over bilateral lung fields. Her abdomen was soft and non-tender with no organomegaly.

Initial blood investigations performed are summarized below in Table 1, and apart from the very marginally elevated ESR value, the rest of the blood investigations appeared to be normal. She also tested negative for human immunodeficiency virus (HIV) and hepatitis virus upon serological screening.

**Table 1:** Basic blood investigations performed on admission WBC: white blood cells; CRP: C-reactive protein; ESR: erythrocyte sedimentation rate; AST: aspartate transaminase; ALT: alanine transaminase; ALP: alkaline phosphatase; APTT: activated partial thromboplastin time; PT/INR: prothrombin time/international normalized ratio

Investigation	Value	Reference range with unit
WBC	7.22 × 10^3^	4–11 × 10^3^ µL
Neutrophils	4.2 × 10^3^	2–7 × 10^3^ µL
Lymphocytes	2.4 × 10^3^	0.8–4 × 10^3 ^µL
Eosinophils	0.06 × 10^3^	0.02–0.5 × 10^3^ µL
Hemoglobin	11.1	11–16 g/dL
Platelets	298 × 10^3^	150 × 10^3^–450 × 10^3^ µL
CRP	2	<6 mg/L
Sodium	141	136–145 mmol/L
Potassium	4.1	3.5–5.3 mmol/L
Serum creatinine	59	45–90 µmol/L
ESR	38	0–30 mm/hour
AST	18	0–37 U/L
ALT	10	7–35 U/L
ALP	116	30–120 U/L
Total bilirubin	0.2	0.2–1.1 mg/dL
APTT	23	21–35 secs
PT/INR	0.9	0.8–1.1

**Figure 1:**
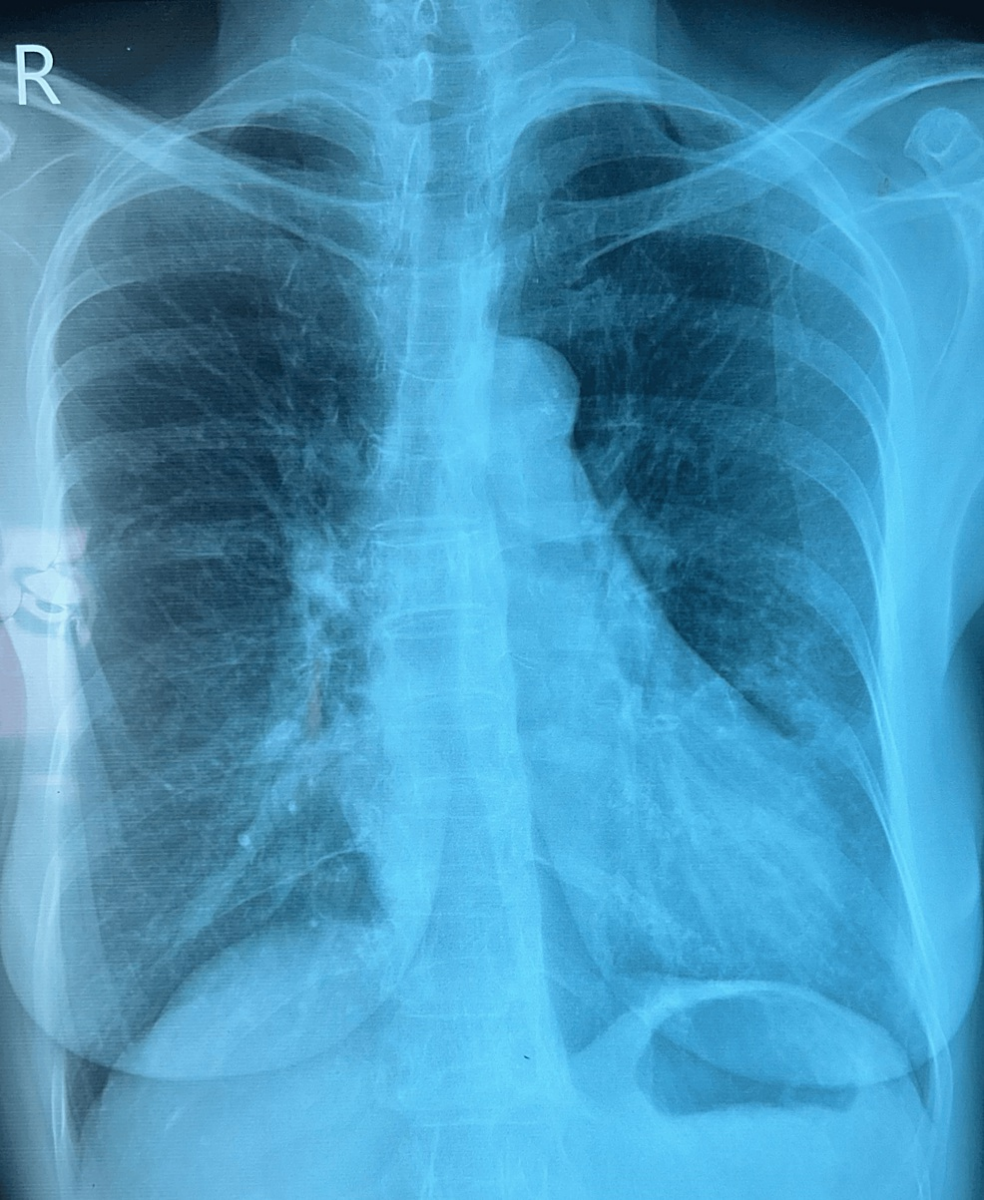
Chest X-ray of the 64-year-old female with intermittent fever, dry cough for one month with recent-onset hemoptysis

A chest X-ray was performed on our patient, which was essentially normal (Figure 1). However, since the patient had significant symptoms and Sri Lanka is an endemic country for tuberculosis, further imaging with a high-resolution CT (HRCT) scan was also planned.

**Figure 2:**
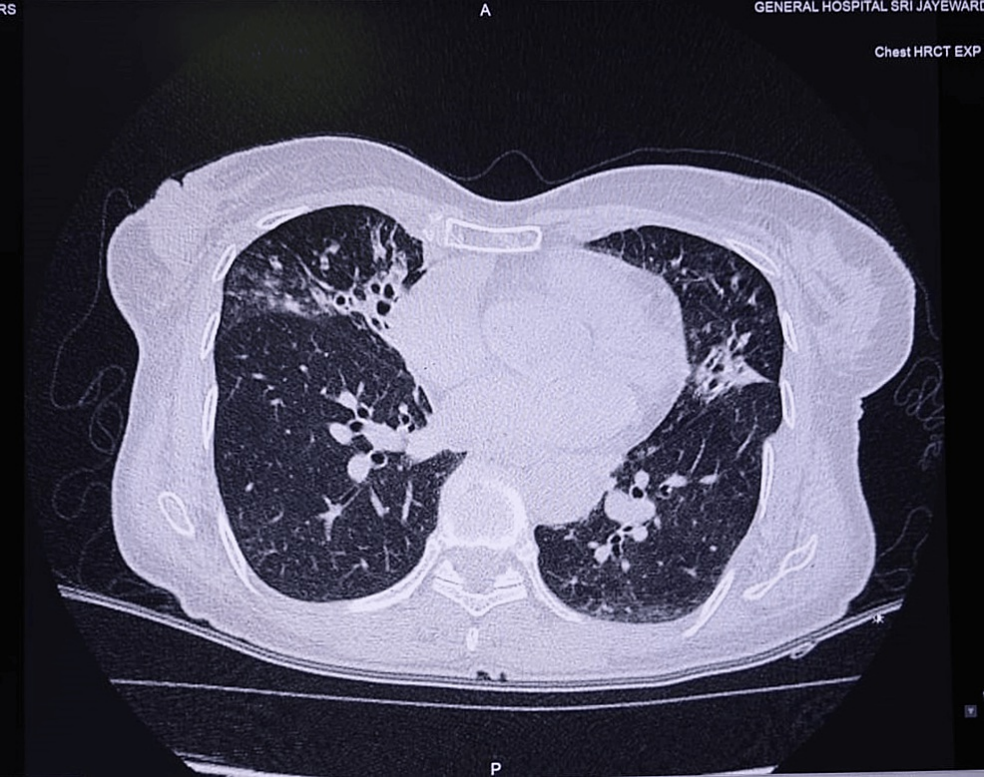
HRCT of the 64-year-old female with intermittent fever and dry cough for one month with recent-onset hemoptysis (axial view) HRCT: high-resolution CT

The HRCT scan revealed that she had bronchiectasis with the associated bronchiolitis in the anterior segment of the right upper lobe and middle lobe of the right lung, and lingula lobe and superior segment of the left lower lobe likely because of MAC infection (Figure 2).

Because of the recent HRCT findings, the Mantoux test, sputum culture, sputum for GeneXpert, and three early morning sputum samples for acid-fast bacilli were sent, all of which yielded negative results. After liaising with the respiratory team, a bronchoscopy was planned and performed, which did not reveal any features suggestive of malignancy or active infection except for some distal yellow thin secretions in the left lingula lobe. The washings done from multiple segments were sent for bacterial, fungal, and tuberculosis cultures as well as GeneXpert and AFB. Out of the abovementioned tests, all were negative, except for the tuberculosis culture that became positive for MAC.

Once the diagnosis was established following the positive sputum culture, the patient was initiated on clarithromycin, ethambutol, and rifampicin drug combination at the clinic level. She was followed up with a regular assessment of her symptoms and lung signs, and she showed a significant response to the treatment.

## Discussion

Mycobacteria are a group of gram-positive, acid-fast bacilli [5]. They are classically known to cause tuberculosis and leprosy, which are caused by Mycobacterium tuberculosis and Mycobacterium leprae, respectively. The genus Mycobacterium contains more than 190 species, and apart from the abovementioned two species, the rest of the Mycobacterium species are collectively known as nontuberculous mycobacteria (NTM) [6]. NTM are a group usually isolated from environmental sources such as water and soil, and unlike Mycobacterium tuberculosis, they are not obligate pathogens. NTM can be broadly classified into four groups based on their growth rates, colony morphology, and pigmentation, and this is known as the Runyon classification; MAC belongs to group 3 according to this classification system [3].

The major clinical syndromes caused by NTM are pulmonary disease, lymphadenitis, cutaneous disease, and disseminated disease [3]. Although infections caused by NTM are classically known to occur in immunocompromised patients, they can sometimes occur in immunocompetent patients as well, and when they do occur, they give rise to pulmonary disease 77% of the time [7].

Pulmonary disease caused by MAC can either result in an upper lobe cavitary form or nodular bronchiectatic form [3]. The upper lobe cavitary form usually occurs in patients with pre-existing lung conditions such as chronic obstructive pulmonary disease, silicosis, and a history of tuberculosis, while the nodular bronchiectatic form often tends to occur in patients with no previous history of lung conditions, and the latter is also known as LWS [3].

LWS is a condition occurring particularly in elderly, thinly built, non-immunocompromised females and is caused by pulmonary infection of MAC. It was first described in 1992, following a case series where elderly females were found to be harboring MAC leading to pulmonary disease, especially involving the lingula lobe and middle lobe of the lung [2]. The other main observation noted in these cases was that this occurred in patients who did not have any predisposing pulmonary diseases. A hypothesis that chronic voluntary cough suppression, especially in female patients, leads to MAC infection in dependent lobes of the lung was brought forward in this case report [2]. Later, it was noted that elderly females with scoliosis, pectus excavatum, and mitral valve prolapse were also at higher risk of developing LWS [8].

There are characteristic radiographic findings that aid in the diagnosis of LWS. On chest X-ray, there may be linear opacities in the middle lobe and lingula lobe, and on CT scan images, bronchiectasis, centrilobular nodules, tree-in-bud nodularity characteristically affecting the middle lobe and lingula lobe can be seen [9]. The tendency of LWS to occur mainly in the middle lobe and lingula lobe of the lung is postulated to be because of the anatomical structure of these lobes where they are more dependent on coughing to clear the secretions [10].

In our patient, there were characteristic HRCT findings of bronchiectasis associated with bronchiolitis in the right upper lobe and middle lobe of the right lung, lingula lobe, and superior segment of the lower lobe of the left lung. The CT findings were the main pointer towards the diagnosis of LWS, which was further supported by the positive culture for MAC. In resource-limited settings, arriving at a diagnosis of LWS is seldom achieved in a patient presenting with hemoptysis mainly because of advanced imaging such as HRCT not being readily available. Usually, in resource-limited settings, patients such as ours who are presenting with mild and non-life-threatening symptoms will often be discharged with a date for HRCT once serious conditions such as tuberculosis are satisfactorily excluded with a tuberculosis culture report to be traced at the clinic level. These patients are frequently lost to follow-up; thus, diagnosis of LWS, which itself is a rare entity, is even rarer in resource-limited settings.

In a patient presenting with hemoptysis, even in the absence of severe symptoms, it is important to investigate thoroughly to arrive at a diagnosis. Especially in a country where tuberculosis is endemic, even if the initial tuberculosis investigations are negative with an initial and apparently normal chest X-ray, employing advanced imaging such as HRCT plays a significant role in arriving at a diagnosis. It is also important to bear in mind that just because a patient is not immunocompromised with no predisposing lung pathologies, the possibility of MAC infection should not be disregarded especially in the setting of supportive HRCT findings.

The standard treatment for MAC infection is macrolides (clarithromycin or azithromycin), rifampicin, and ethambutol combination [4]. In cases of nonresponse to the abovementioned medical management, surgical interventions with selective pulmonary resection can be considered in a small group of patients [11]. The differentiation between MAC and tuberculosis is essential as the treatment options for the two conditions are quite different.

## Conclusions

LWS is a rare entity of pulmonary infection of MAC that occurs in immunocompetent patients, particularly elderly females. The diagnosis is mainly based on radiographical findings where features of bronchiectasis associated with bronchiolitis can be visualized, especially affecting the middle lobe and the lingula lobe of the lung. Further tests to exclude tuberculosis and other immunodeficient conditions should be conducted before arriving at the diagnosis of LWS. The diagnosis can be reinforced by the positive culture of MAC. Once the diagnosis is confirmed, the patient should be started on a combination of macrolide, rifampicin, and ethambutol and should be followed up regularly to assess the response to treatment. Especially in countries where a considerable number of pulmonary tuberculosis cases are encountered regularly, prompt diagnosis of LWS is important as the treatment regime is different in both conditions. This case report emphasizes the significance of chest imaging (HRCT) in a patient who was initially thought to be having tuberculosis, later confirmed to be a case of LWS.

## References

[1] Akram SM, Attia FN (2023). Mycobacterium avium complex. StatPearls Publishing.

[2] Reich JM, Johnson RE (1992). Mycobacterium avium complex pulmonary disease presenting as an isolated lingular or middle lobe pattern. The Lady Windermere syndrome. Chest.

[3] Koh WJ, Kwon OJ, Lee KS (2002). Nontuberculous mycobacterial pulmonary diseases in immunocompetent patients. Korean J Radiol.

[4] Rao R, Sheshadri S, Patil N, Rao K, Arivazhahan A (2016). Lady Windermere syndrome: a very rare entity in Indian medical scenario. J Clin Diagn Res.

[5] Cook GM, Berney M, Gebhard S, Heinemann M, Cox RA, Danilchanka O, Niederweis M (2009). Physiology of mycobacteria. Adv Microb Physiol.

[6] (2024). Atypical mycobacterial diseases. https://emedicine.medscape.com/article/1105570-overview.

[7] Henkle E, Winthrop KL (2015). Nontuberculous mycobacteria infections in immunosuppressed hosts. Clin Chest Med.

[8] Cataño JC, Porras Mancilla JP (2022). Lady Windermere syndrome. Postgrad Med J.

[9] Gaillard F (2024). Lady Windermere syndrome. https://radiopaedia.org/articles/lady-windermere-syndrome.

[10] Dhillon SS, Watanakunakorn C (2000). Lady Windermere syndrome: middle lobe bronchiectasis and Mycobacterium avium complex infection due to voluntary cough suppression. Clin Infect Dis.

[11] Yu JA, Pomerantz M, Bishop A, Weyant MJ, Mitchell JD (2011). Lady Windermere revisited: treatment with thoracoscopic lobectomy/segmentectomy for right middle lobe and lingular bronchiectasis associated with non-tuberculous mycobacterial disease. Eur J Cardiothorac Surg.

